# Theoretical Modeling of Multi-Channel Intracavity Spectroscopy Technology Based on Mode Competition in Er-Doped Fiber Ring Laser Cavity

**DOI:** 10.3390/s20092539

**Published:** 2020-04-29

**Authors:** Haiwei Zhang, Liangcheng Duan, Yan Zhao, Lifang Xue, Pengbo Jiang, Jun Liu, Yangbo Bai, Wei Shi, Jianquan Yao

**Affiliations:** 1 Engineering Research Center of Optoelectronic Devices and Communication Technology (Ministry of Education), Tianjin Key Laboratory of Film Electronic and Communication Device, School of Electrical and Electronic Engineering, Tianjin University of Technology, Tianjin 300384, China; zhanghaiwei@email.tjut.edu.cn (H.Z.); 15811012070@163.com (L.X.); cloudlj@163.com (J.L.); baiyangbo_tj@163.com (Y.B.); 2 Institute of Laser and Optoelectronics, Key Laboratory of Optoelectronics Information Science and Technology (Ministry of Education), School of Precision Instrument and Optoelectronics Engineering, Tianjin University, Tianjin 300072, China; dlc@tju.edu.cn (L.D.); shiwei@tju.edu.cn (W.S.); jqyao@tju.edu.cn (J.Y.); 3 Laser Institute, Qilu University of Technology (Shandong Academy of Sciences), Jinan 250014, China; jiangpengbo@sdlaser.cn

**Keywords:** intracavity spectroscopy technology, multiplexing technology, numerical simulation, optical fiber laser, laser excitation

## Abstract

An analytical model for analyzing multi-channel intracavity spectroscopy technology (ICST) is established based on rate equations of Er-doped fiber laser. With the consideration of the amplified spontaneous emission, how the mode competition influences the iterative process for a stable output is analyzed. From the perspective of iterative times, the sensitivity-enhanced mechanism of the ICST is explained. Moreover, the theoretical modeling is employed to analyze the role that the mode-competition effect plays in switching the sensing channel automatically. It is demonstrated that, owing to the mode-competition effect in the laser cavity, the modulation of the cavity loss can be used to tune the sensing channel automatically. Furthermore, our proposed theoretical modeling is verified using a multi-channel ICST sensing system. It is indicated that the calculated estimates agree well with those data from the experimental absorption spectra. The principle will play a significant role in realizing the multiplexing of ICST.

## 1. Introduction

Intracavity spectroscopy technology (ICST) was originally used to enhance the sensitivity of the sensing system through placing the analyte in a laser resonator to increase the amount of interactions between the laser and the analyte [1]. Such a technology has been playing a significant role in detecting the concentration of molecules such as acetylene, methane, and ammonia [2,3]. Compared with those single-pass sensing system, the laser cavity of ICST is favored by researchers owing to its other unique advantages such as mode competition and higher light intensity in the resonator for improving the sensitivity. Recently, Yang et al. improved the sensitivity of a low-concentration gas sensor more than six-fold via the mode-competition effect in a dual-wavelength ring fiber laser [4]. Wang et al. realized a sensitivity-enhanced refractive-index sensor using the gain competition in a linear-cavity dual-wavelength erbium-doped fiber laser [5]. Considering that the light intensity in the resonator can be made higher than the single-pass laser output, ICST can be employed to substantially improve the sensitivity via the photoacoustic or photothermal spectroscopy. Zhang et al. realized a gas sensor with a sensitivity of sub-ppmv combined ICST with the photoacoustic spectroscopy [6]. Zhao et al. offered a solution to achieve a sub-ppbv sensitivity intracavity acetylene sensor by combining ICST with the photothermal spectroscopy technology [7]. Moreover, the broad-wavelength tunability of the intracavity laser (ICL) expands its applications in the measurements of the magnetic field [8], temperature [9], and refractive index [10], through analyzing the high signal-to-noise-ratio wavelength shifts.

However, there is no denying that the ICST system is really more complex in comparison with those passive sensing systems. Such a disadvantage leads to a higher cost only for a single-point measurement. Therefore, researchers are focusing their attentions on finding more effective approaches to realize multipoint sensing, so as to decrease the cost imposed on each access point. It is demonstrated that the wavelength tunable property gives it the capability to construct a sensor network via the wavelength multiplexing technology. Liu et al. realized an intracavity sensor system using wavelength-swept technique. It had the capability of multi-channel detection by the aid of an optical switch [11]. Zhang et al. proposed an intracavity absorption sensor network based on a mode-locking principle by addressing mode-locking frequencies. In the system, a pulse generator and Mach–Zehnder modulator are needed to realize multipoint compared with the traditional ICST system [12]. Though those systems can achieve multipoint measurements, the commercial active components used in their systems are expensive. Moreover, time-synchronization control between the active devices and scanning process is desired for eliminating the cross-talks among the sensing channels. In order to overcome the above problems that appear in multiplexing the ICST system, several multipoint systems have been proposed to increase the sensing channel via the mode competition in the ring laser cavity [13,14]. Owing to the mode-competition effect, the sensing channel can be switched automatically along with a wavelength-swept filter, so as to make the time-synchronization control system absent.

The reported literature on multipoint ICST mainly focuses on the experimental results, and the theoretical modeling for analyzing their multiplexing characteristics has never been reported. Moreover, the previously reported analyses for ICST are mainly based on the simplified formulas, which make them available only for single-point sensing system [15,16,17]. Considering that amplified spontaneous emission (ASE) has a significant influence on the gain of the signal [18,19,20], the models that ignore ASE are inapplicable to analyze a multi-channel ICST system based on the mode-competition effect induced by the gain difference in the laser cavity. In this paper, we first propose a theoretical modeling for multipoint ICST though introducing a cavity-loss factor into the rate equations. Then, the reason for enhancing sensitivity is given by solving the modified rate equations with ASE. Finally, the multi-channel sensing principle based on mode competition is proposed and the effectiveness of the numerical prediction is verified though an experiment.

## 2. Theoretical Modeling and Solution Methods

### 2.1. Theoretical Modeling

**Figure 1:**
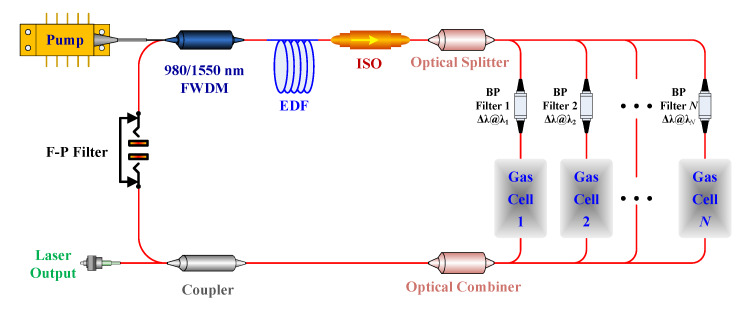
Schematic principle of the sensing system. EDF, Er-doped fiber; ISO, isolator; FWDM, filter wavelength division multiplexing; F–P, Fabry–Perot; BP, band-pass.

For the ICST system illustrated in Figure 1, it can be regarded as a tunable Er-doped fiber (EDF) laser. Its characteristics are usually analyzed through a three-level system that consists of a ground level (^4^*I*_15/2_), metastable level (^4^*I*_13/2_), and pump level (^4^*I*_11/2_). Therefore, the population densities *N*_1_, *N*_2_, and *N*_3_ in the ground, metastable, and excited state, respectively, can be described via the differential Equations (1)–(3) [21,22,23]:

(1) $$\begin{array}{l} \frac{\partial N_{1}(z,t)}{\partial t}=-\left[\frac{\sigma_{sa}\Gamma_{s}\lambda_{s}}{hcA}[P_{s}^{+}(z,t)+P_{s}^{-}(z,t)]+\sum_{i=1}^{x}\frac{\sigma_{sa}(\lambda_{i})\cdot \Gamma_{s}\cdot \lambda_{i}}{hcA}\left[P_{ase}^{+}(z,t,\lambda_{i})+P_{ase}^{-}(z,t,\lambda_{i})\right]\right]N_{1}\\ -\;\frac{\sigma_{pa}\Gamma_{p}\lambda_{p}}{hcA}\left[P_{p}^{+}(z,t)+P_{p}^{-}(z,t)\right]N_{1}+\frac{\sigma_{pe}\Gamma_{p}\lambda_{p}}{hcA}\left[P_{p}^{+}(z,t)+P_{p}^{-}(z,t)\right]N_{3}\\ +\;\left[\frac{\sigma_{se}\Gamma_{s}\lambda_{s}}{hcA}\left[P_{s}^{+}(z,t)+P_{s}^{-}(z,t)\right]+\sum_{i=1}^{x}\frac{\sigma_{se}(\lambda_{i})\cdot \Gamma_{s}\cdot \lambda_{i}}{hcA}\left[P_{ase}^{+}(z,t,\lambda_{i})+P_{ase}^{-}(z,t,\lambda_{i})\right]+\frac{1}{\tau_{21}}\right]N_{2} \end{array}$$

(2) $$\begin{array}{l} \frac{\partial N_{2}(z,t)}{\partial t}=\left[\frac{\sigma_{sa}\Gamma_{s}\lambda_{s}}{hcA}[P_{s}^{+}(z,t)+P_{s}^{-}(z,t)]+\sum_{i=1}^{x}\frac{\sigma_{sa}(\lambda_{i})\cdot \Gamma_{s}\cdot \lambda_{i}}{hcA}[P_{s}^{+}(z,t)+P_{s}^{-}(z,t)]\right]\cdot N_{1}+\frac{1}{\tau_{32}}N_{3}\\ -\left[\frac{\sigma_{se}\Gamma_{s}\lambda_{s}}{hcA}[P_{s}^{+}(z,t)+P_{s}^{-}(z,t)]+\sum_{i=1}^{x}\frac{\sigma_{se}(\lambda_{i})\cdot \Gamma_{s}\cdot \lambda_{i}}{hcA}[P_{s}^{+}(z,t)+P_{s}^{-}(z,t)]+\frac{1}{\tau_{21}}\right]\cdot N_{2} \end{array}$$

where *P* is defined as the power. The subscripts *s*, *p*, and *ase* represent the signal, pump, and ASE, respectively, while the superscripts + and − represent that the signal has a co- or counter-propagation direction with pump and ASE, respectively. Γ*_p_* represents the fraction of the pump power coupled into the active core, and Γ*_s_* represents the transverse overlap between the laser beam intensity and dopants concentration profiles. *λ_i_* is denoted as the *i*th wavelength in the ASE. The absorption cross sections of the pump and signal are denoted by *σ_pa_* and *σ_sa_*, respectively, while *σ_pe_* and *σ_se_* designates the emission cross sections of the pump and signal, respectively. *A* is the area of the active fiber core. *τ*_21_ and *τ*_32_ are denoted as the decay times from metastable level to ground level and from pump level to metastable level, respectively. *h* and *c* are the Planck’s constant and the velocity of the light in vacuum. The population densities in different levels satisfy the following expression:

(3) $$N_{1}+N_{2}+N_{3}=N_{t},$$

where *N_t_* is the total active ions density.

Meanwhile, the power transfer equations for the EDF laser are given in (4) and (5):

(4) $$\pm \frac{\partial P_{p}^{\pm }(z,t)}{\partial z}+\frac{1}{v_{p}}\frac{\partial P_{p}^{\pm }(z,t)}{\partial t}=P_{p}^{\pm }(z,t)\cdot \Gamma_{p}\left(\sigma_{pe}N_{3}-\sigma_{pa}N_{1}\right)-\alpha_{p}P_{p}^{\pm }(z,t),$$

(5) $$\pm \frac{\partial P_{s}^{\pm }(z,t)}{\partial z}+\frac{1}{v_{s}}\frac{\partial P_{s}^{\pm }(z,t)}{\partial t}=P_{s}^{\pm }(z,t)\cdot \Gamma_{s}\left(\sigma_{se}N_{2}-\sigma_{sa}N_{1}\right)-\alpha P_{s}^{\pm }(z,t).$$

In order to provide flexibility in choice of the number of signals and their wavelengths for sweeping the absorption peaks of the gases, the influencing factor induced by the ASE should also be considered in the model:

(6) $$\begin{array}{l} \pm \frac{\partial P_{ase}^{\pm }(z,t,\lambda_{i})}{\partial z}+\frac{1}{v}\frac{\partial P_{ase}^{\pm }(z,t,\lambda_{i})}{\partial t}=P_{ase}^{\pm }(z,t,\lambda_{i})\cdot \Gamma_{s}\left(\sigma_{se}N_{2}-\sigma_{sa}N_{1}\right)-\alpha P_{ase}^{\pm }(z,t,\lambda_{i})\\ +2\sigma_{se}(\lambda_{i})N_{2}(z,t)\Gamma_{s}\frac{hc^{2}}{\lambda_{i}^{3}}\Delta \lambda \end{array}$$

where *v_p_*, *v_s_*, and *v* are the group velocities of the pump, signal, and ASE in the fiber laser, respectively. Δ*λ* is defined as the wavelength interval in the whole emission-spectrum. *α_p_* is the internal loss of the pump in the laser cavity. The loss term *α* is determined by both the loss of the signal and the absorption of the gas, which can be expressed as follows:

(7) $$\alpha =\alpha_{s}+\alpha_{a}.$$

Here, *α_s_* is the internal loss of the signal that induced by the tunable Fabry–Perot filter (F–P filter), band-pass filter (BP filter), and the absorption of the active fiber. Meanwhile, *α_a_* represents the additional cavity loss determined by the gas-absorption intensity and the number of times.

### 2.2. Theoretical Solutions

In steady-state conditions where ∂*N*/∂*t* = 0 and ∂*P*/∂*t* = 0, the differential Equations (1)–(6) can be solved by using the finite difference method (FDM). Considering that the ICL illustrated in Figure 1 is based on a forward pumping scheme, the above rate and power transfer equations should satisfy the following initial-boundary conditions:

(8) $$P_{p}^{+}(0)=P_{1},\;P_{p}^{-}(L)=0,$$

(9) $$P_{ase}^{+}(0,\lambda_{i})=P_{a},\;P_{ase}^{-}(L,\lambda_{i})=0,\;i=1,2,\ldots ,x,\;x\neq s,$$

(10) $$P_{s}^{+}(0)=P_{2},\;P_{s}^{-}(L)=0.$$

**Figure 2:**
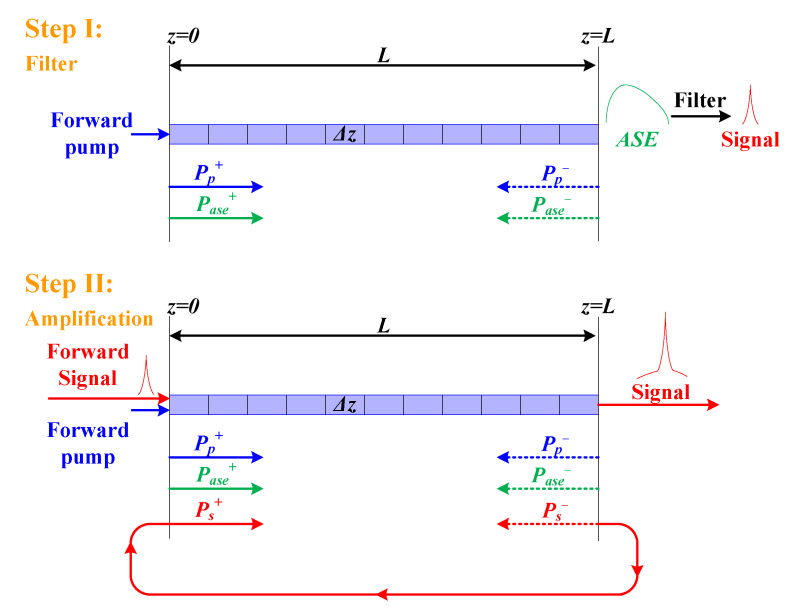
Calculation process. ASE, amplified spontaneous emission.

Here, *L* represents the length of the active fiber. It is obvious that *P*_1_ can be obtained from the pump source. As for the value of initial-boundary *P*_2_ related to the signal, its value should be considered according to the process for generating the signal. *P*_a_ is determined by the transmission of the F–P filter. As displayed in Figure 2, the calculation process is divided into the filter and amplification steps to confirm the initial-boundary value of the input signal, respectively.

In the filter step, there is no signal injected into the active fiber. Therefore, the value of initial-boundary *P*_2_ is equal to zero. In this process, the signal is regarded as one wavelength among the ASE spectrum. So, the power transfer equations describing the signal are set to be differential Equation (6) instead of (5). Then, decomposing the active fiber into *L*/Δ*z* discrete elements and solving Equations (1)–(4) and (6) though the FDM, we can obtain the power of all the wavelengths in the ASE spectrum at the end of the active fiber under the initial-boundary conditions (8)–(10). Owing to the function of the BP filter and the F–P filter, the power of the signal corresponding to the value of $P_{ase}^{+}(L,\lambda_{s})$ can be achieved from the calculated ASE spectrum.

Then, the rest of the analyses of the model can be calculated like an Er-doped fiber amplifier. In particular, the filtered signal will be injected and amplified iteratively until the system has a stable output. Now, the initial-boundary condition for the calculation is expressed as follows:

(11) $$P_{s}^{+}(0)=P_{ase}^{+}(L,\lambda_{s})\cdot \left(1-\alpha -R\right),\;P_{s}^{-}(L)=0.$$

Here, *R* represents the ratio that the coupler extracts the power output of the cavity. Similarly, the active fiber is decomposed into *L*/Δ*z* discrete elements and the rate Equations (1)–(6) under the initial boundary condition of (8), (9), and (11) is solved via the FDM. We cannot eventually achieve the distribution of the power along the fiber and the lasing spectrum with the ASE until the gain of the signal is equal to the loss of the cavity and the ICL has a stable output. Therefore, an iterative process is needed for the ICL to have a stable output.

**Figure 3:**
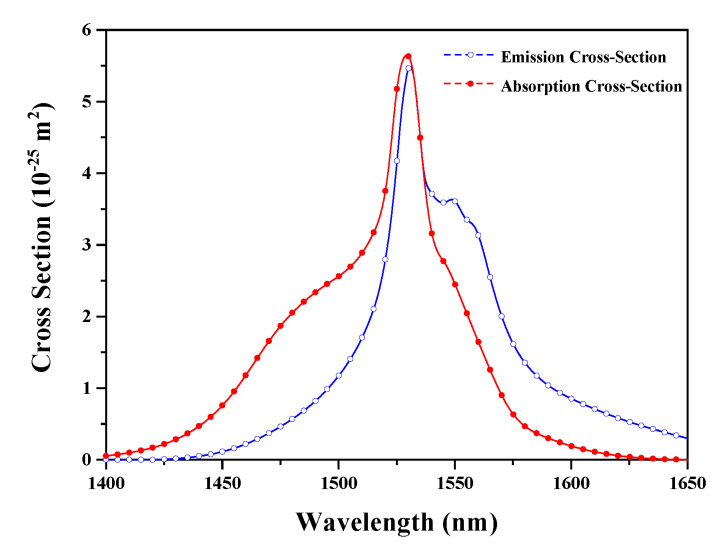
Emission and absorption cross-sections of the EDF [24].

**Figure 4:**
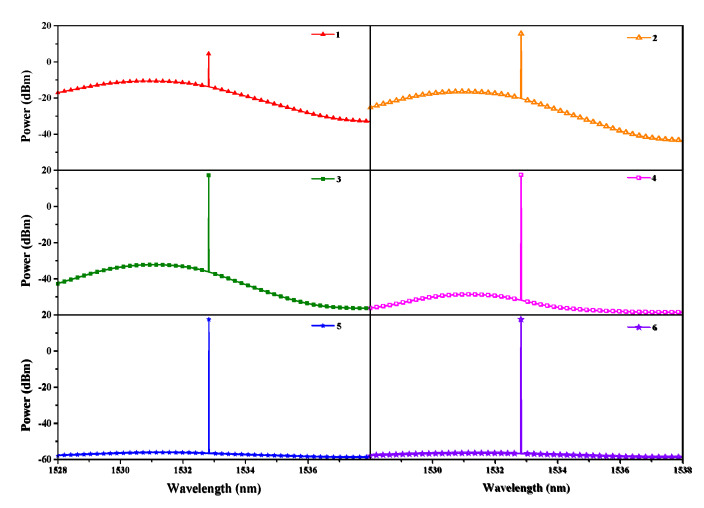
Spectra of the output laser in each iterative process.

Using the parameters listed in Table 1 and the cross-sections indicated in Figure 3, we can achieve the spectra of the output laser in each iterative process, as illustrated in Figure 4. During the iterative process, the power of the output laser is enhanced gradually, while the ASE is restrained correspondingly. Such a phenomenon is attributed to the mode-competition effect in the resonator, which can make the lower-loss signal extract a more conversed population and achieve a higher gain. It can also be found out that the whole amplification process displayed in Figure 2 will duplicate at least five times to make the gain equal to the cavity loss and obtain a stable output power, as the processes indicated in Figure 4.

From the iterative process illustrated in Figure 4, it is found out that the ICST system needs more interaction times to obtain a stable output compared with the single-pass sensing systems. As a result, such an iterative process increases the interactions between the laser and the analyte and causes the ICST system have an enhanced sensitivity.

**Figure 5:**
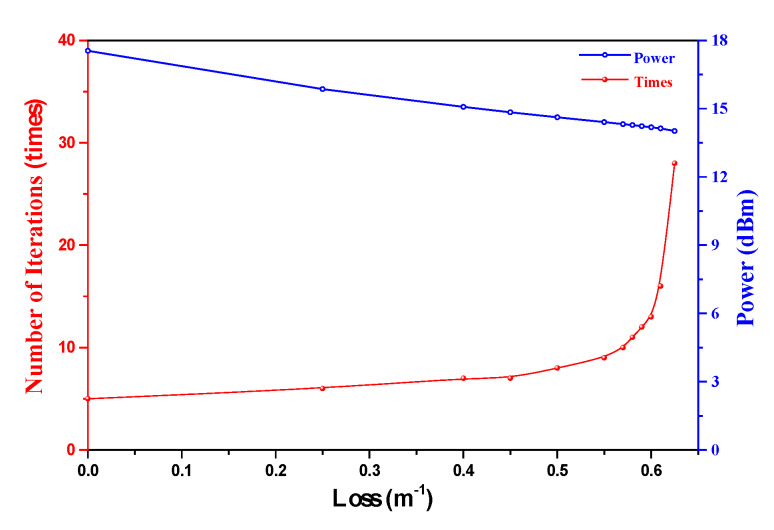
Iterative times and output power versus additional loss.

When the additional loss applied to the signal increases from 0 to 7 × 10^−1^ m^−1^, the iterative times needed for a stable output and the output power of the ICST system versus the cavity loss are illustrated in Figure 5.

In the process of the additional loss changing from 0 to 7 × 10^−1^ m^−1^, the output power decreases because the increased cavity loss decreases the gain and increases the pump threshold. As a result, more iterative times are needed to make the gain equal to the cavity loss to achieve a stable output. Therefore, making the ICL operate close to its threshold can increase the interaction times, thus enhancing the sensing sensitivity. Such a calculation result agrees well with the previously reported conclusion that the sensitivity can be enhanced when the ICST system operates close to its pump threshold [15,16,17].

## 3. Multi-Channel Sensing Principle and Calculation

Owing to the mode-competition effect in the resonator, the higher gain signal will become increasingly higher, and the lower one will be restrained automatically. Therefore, the multi-channel sensing system illustrated in Figure 1 is expected to be used to realize the multi-channel sensing considering the influence induced by the mode-competition effect on the spectra shown in Figure 4.

**Figure 6:**
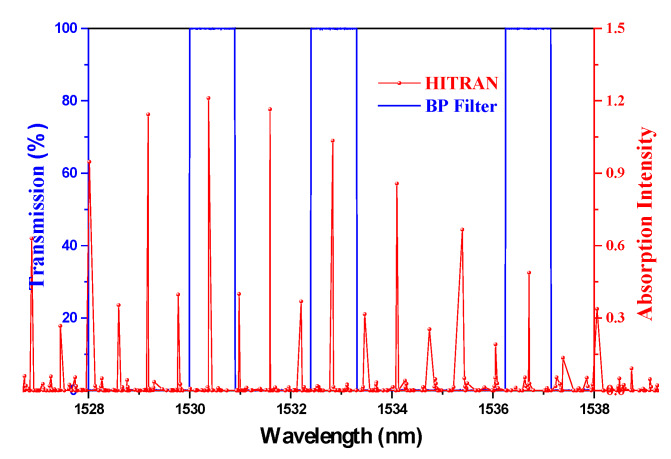
Relationship between the BP filter and the absorption peaks of C_2_H_2_.

C_2_H_2_ is chosen as the analyte to explain the sensing principle. According to its absorption peaks and the reported database, the wavelength parameter *λ_s_* in the rate equations is confirmed to be 1530.37, 1532.83, and 1536.71 nm, respectively [25]. For the purpose of decreasing the channel crosstalk, the transmission linewidth of the BP filter is chosen as narrow as possible to only cover one of the absorption peaks. The relationship between the narrow-band filters and the absorption peaks are illustrated in Figure 6.

**Figure 7:**
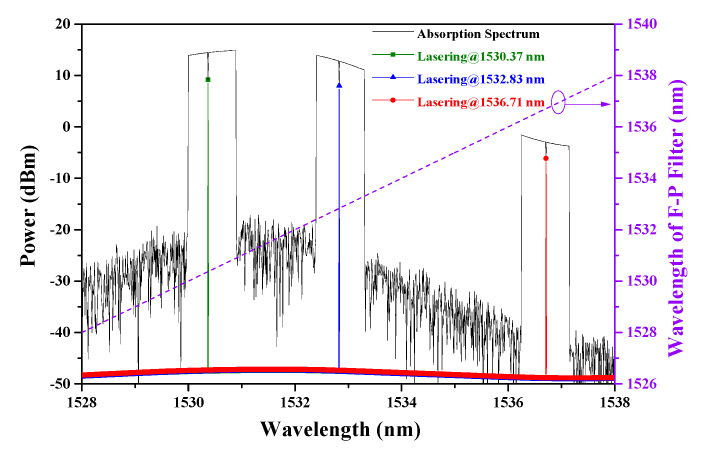
Calculated absorption spectra and laser spectra via tuning the F–P filter.

Using the parameters illustrated in Figure 6 and the calculation method mentioned above, we obtain the spectra of the signal in the absorption peaks and the swept spectra of the sensing system in the whole tuning range, as shown in Figure 7.

While the operating wavelength of the F–P filter changes from 1528 to 1538 nm linearly, it can be found that the ICL will have three working bands that are coincident with the BP filters. It should be announced that the working bands can be switched automatically during the calculation even though there are no the active devices such as the optical switch, Mach–Zehnder modulator, or pulse generator. Combined with the iterative process illustrated in Figure 4, we can attribute the automatic switching function to the mode-competition effect in the ring laser cavity. Therefore, it is feasible for a sensing system based on ICST to achieve tunable sensing channels only through the modulation of the cavity loss.

When the gas cell is filled with C_2_H_2_, there is a power drop-off in each working band owing to the cavity loss induced by the absorption of C_2_H_2_. Considering that such a cavity loss corresponds to the concentration of the gas, we can achieve the gas-concentration sensing via exploring the relationship between the drop-off of the power and the concentration.

Therefore, for the sensing system illustration in Figure 1, the switch process can be completed automatically only using the F–P filter to control the cavity loss instead of using the optical switch owing to the mode-competition effect in the fiber ring laser cavity. Therefore, it causes the system to have a good synchronism without extra time-synchronization control. In addition, the concentration of the gas in different channels can be distinguished automatically though assigning wavelength to each detection channel in advance.

## 4. Experiments and Results

**Figure 8:**
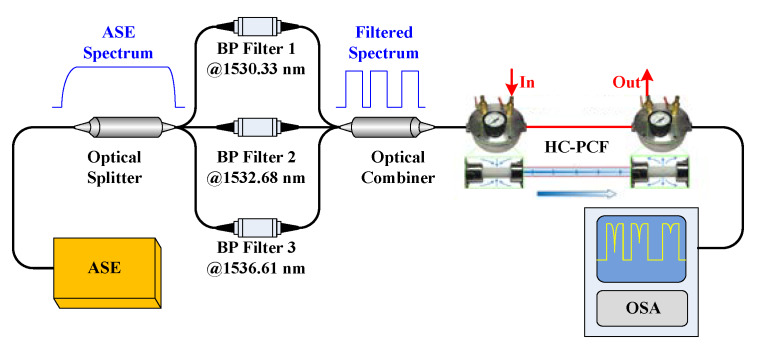
Passive single-pass sensing system. HC–PCF, hollow-core photonic crystal fiber; OSA, optical spectrum analyzer.

In order to confirm the loss factor *α_a_* and realize the numerical calculation, we established the experimental setup shown in Figure 8 to obtain the absorption intensity in the passive sensing situation. The system consists of an ASE light source, an optical splitter, three BP filters, an optical combiner, an optical spectrum analyzer (OSA), as well as a gas cell formed by 1 m long hollow-core photonic crystal fiber (HC–PCF), both ends of which are connected with the single mode fiber through two bare-fiber adapters.

The operation wavelengths of the BP filters are confirmed according to the absorption peaks in Figure 6 and international telecommunications union (ITU) standards [13]. As the experimental setup illustrated in Figure 8, the spectrum of the ASE is divided into three narrow passbands, which are assigned to measure the absorption intensities of the gas with different concentrations.

**Figure 9:**
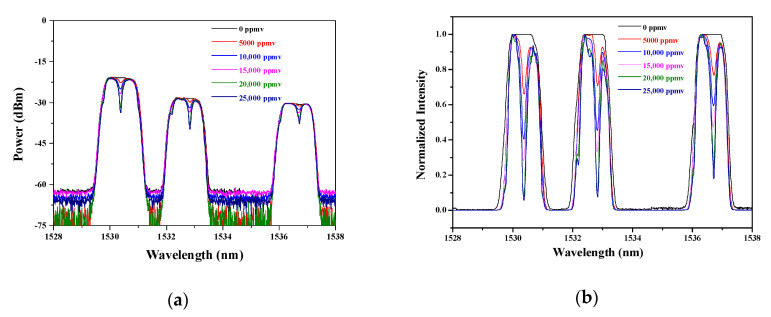
(**a**) Absorption spectra with different concentrations; (**b**) absorption spectra with normalized intensity.

When the concentration of C_2_H_2_ changes from 0 to 25,000 ppmv, the measured absorption spectra are displayed in Figure 9a. Considering that the factor that plays a role in affecting the additional cavity-loss *α_a_* is the variation of power, each absorption peak locating in the BP filter should be normalized so as to get the cavity loss. The calculated results are illustrated in Figure 9b. Then, the normalized intensity can be employed to calculate the characteristics of the ICL via solving the power transfer equations.

**Figure 10:**
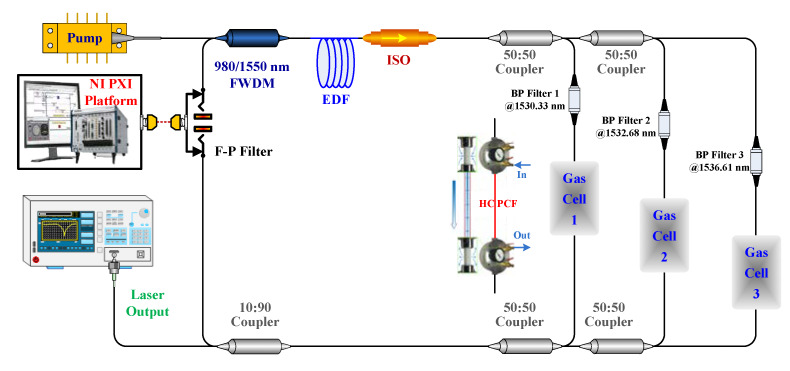
Multipoint sensing system based on intracavity spectroscopy technology (ICST).

To verify the feasibility of our proposed theoretical modeling for the multi-channel ICST sensing system, we established an experimental setup for multipoint sensing based on ICST as it is illustrated in Figure 10, which is same as our reported setup in [13].

**Figure 11:**
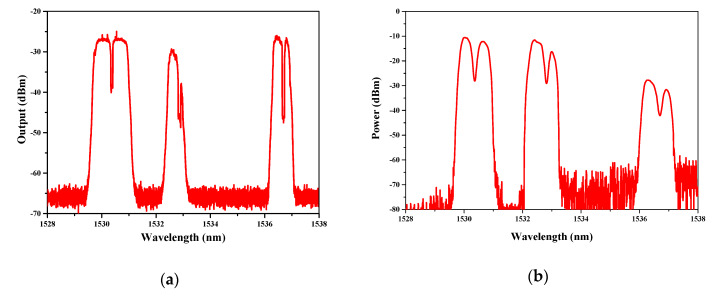
(**a**) Experimental absorption spectra with 10,000 ppmv C_2_H_2_; (**b**) calculated absorption spectra with normalized intensity.

When the gas cells are filled with 10,000 ppmv C_2_H_2_ and the F–P filter is tuned from 1528 to 1538 nm, the absorption spectra can be obtained, as illustrated in Figure 11a.

In order to verify the feasibility of our proposed theoretical modeling for analyzing the multi-channel ICST sensing system, we calculate the absorption spectra using the normalized absorption spectra illustrated in Figure 9b. The numerical result is illustrated in Figure 11b. Compared with Figure 11a,b, it can be found out that both of them have three working bands. Both the bandwidth and absorption intensity of the scanned spectra around 1530.37 nm match well with each other. The difference of the spectra around 1532.83 and 1536.71 nm is mainly induced by the bandwidth of the BP filter. Though the difference of the BP filter makes the bandwidth and absorption intensity different, the experimental results can still be used to verify the validity of the theoretical modeling and calculation method, because the experiment and simulation have the same parameter only around 1530.37 nm.

Moreover, from the figure, we can also find that the linewidth of the BP filter may have an effect on the absorption intensity, so as to have an influence on the sensitivity. Considering that the narrower bandwidth of the BP filter causes the system to have a higher absorption intensity, narrowing linewidth is expected to have a benefit in enhancing the sensitivity.

## 5. Conclusions

This paper presents a theoretical modeling for the multi-channel ICST system through introducing the ASE and the cavity-loss factor into the rate equations. According to the lasing spectra from the ICL, it needs a certain number of resonance times for the ICL to have a stable output. Such an iterative process can be employed to explain how the ICST enhances the sensitivity compared with the single-pass sensing system, and it can also be used to analyze the reason for the sensitivity-enhanced phenomenon around the pump threshold. Owing to the mode-competition effect in the laser cavity, the modulation of the cavity loss can be used to switch the sensing channel automatically through tuning the F–P filter linearly. Finally, we compare the calculated results with the experimental results. It is indicated that the numerical absorption spectra match well with the measured scanned spectra. Our proposed theoretical modeling can be expected to provide a fundamental basis for the wavelength multiplexing of ICST.

## Figures and Tables

**Table 1:** Intracavity laser (ICL) parameter used in the calculation.

Parameter	Value	Parameter	Value
*λ_p_*	980 nm	*τ*_21_	10 ms [21]
*λ_s_*	1532.83 nm	*τ*_32_	1 ns [21]
*λ_i_*	1525−1650 nm	Γ*_p_*	0.85
Δ*λ*	0.01 nm	Γ*_s_*	0.85
*σ_pa_*	1.86 × 10^−25^ m^2^	*σ_sa_*	Figure 3
*σ_pe_*	0.42 × 10^−25^ m^2^	*σ_se_*	Figure 3
*N_t_*	5.4 × 10^24^ m^3^	*α_a_*	0.1 × 10^−1^ m^−1^
*A*	7.1 × 10^−11^ m^2^	*α_p_*	3 × 10^−1^ m^−1^
*h*	6.626 × 10^−34^ J·s	*α_s_*	3 × 10^−1^ m^−1^
*c*	3 × 10^8^ m/s	*R*	0.01

## Abbreviations

These abbreviations contain the list of acronyms used in this manuscript.

*A*	area of the active fiber core
ASE	amplified spontaneous emission
EDF	Er-doped fiber
*c*	velocity of the light in vacuum
*h*	Planck’s constant
ICST	intracavity spectroscopy technology
ICL	intracavity laser
*L*	length of the active fiber
*N*_1_	population densities in the ground state
*N*_2_	population densities in the metastable state
*N*_3_	population densities in the excited state
N_t_	total active ions density
$P_{ase}^{+}$	power of ASE with co-propagation direction
$P_{ase}^{-}$	power of ASE with counter-propagation direction
$P_{p}^{+}$	power of pump with co-propagation direction
$P_{p}^{-}$	power of pump with counter-propagation direction
$P_{s}^{+}$	power of signal with co-propagation direction
$P_{s}^{-}$	power of signal with counter-propagation direction
*v*	group velocities of the ASE
*v_p_*	group velocities of the pump
*v_s_*	group velocities of the signal
*α_a_*	additional cavity loss
*α_p_*	internal loss of the pump
*α_s_*	internal loss of the signal
Γ*_p_*	fraction of the pump power coupled into the active core
Γ*_s_*	transverse overlap between the laser beam intensity and dopants concentration profiles
Δ*λ*	wavelength interval
Δ*z*	length interval
*λ_i_*	the *i*th wavelength in the ASE
*σ_pa_*	absorption cross sections of the pump
*σ_pe_*	emission cross sections of the pump
*σ_sa_*	absorption cross sections of the signal
*σ_se_*	emission cross sections of the signal
*τ*_21_	decay times from metastable level to ground level
*τ*_32_	decay times from pump level to metastable level
